# Monitoring of Mass Distribution Interventions for Trachoma in Plateau State, Nigeria

**DOI:** 10.1371/journal.pntd.0001995

**Published:** 2013-01-10

**Authors:** Elizabeth A. Cromwell, Jonathan D. King, Scott McPherson, Falam N. Jip, Amy E. Patterson, Aryc W. Mosher, Darin S. Evans, Paul M. Emerson

**Affiliations:** 1 The Carter Center, Atlanta, Georgia, United States of America; 2 Johns Hopkins Bloomberg School of Public Health, Baltimore, Maryland, United States of America; 3 The Carter Center Nigeria, Jos, Nigeria; University of California San Diego School of Medicine, United States of America

## Abstract

Mass drug administration (MDA) with antibiotics is a key component of the SAFE strategy for trachoma control. Guidelines recommend that where MDA is warranted the whole population be targeted with 80% considered the minimum acceptable coverage. In other countries, MDA is usually conducted by salaried Ministry of Health personnel (MOH). In Plateau State, Nigeria, the existing network of volunteer Community Directed Distributors (CDD) was used for the first trachoma MDA. We conducted a population-based cluster random survey (CRS) of MDA participation to determine the true coverage and compared this to coverage reported from CDD registers. We surveyed 1,791 people from 352 randomly selected households in 24 clusters in three districts in Plateau State in January 2011, following the implementation of MDA. Households were enumerated and all individuals present were asked about MDA participation. Household heads were questioned about household-level characteristics and predictors of participation. Individual responses were compared with the CDD registers. MDA coverage was estimated as 60.3% (95% CI 47.9–73.8%) by the survey compared with 75.8% from administrative program reports. CDD registration books for comparison with responses were available in 19 of the 24 clusters; there was a match for 658/682 (96%) of verifiable responses. CDD registers did not list 481 (41.3%) of the individuals surveyed. Gender and age were not associated with individual participation. Overall MDA coverage was lower than the minimum 80% target. The observed discrepancy between the administrative coverage estimate from program reports and the CRS was largely due to identification of communities missed by the MDA and not reported in the registers. CRS for evaluation of MDA provides a useful additional monitoring tool to CDD registers. These data support modification of distributor training and MDA delivery to increase coverage in subsequent rounds of MDA.

## Author Summary

The World Health Organization recommends that mass drug administration for trachoma control reach a minimum of 80% of the target population. Previous evaluations of MDA coverage have demonstrated that administrative reports can bias coverage estimates. A survey of participation in mass drug administration for trachoma control was implemented in three districts in Plateau State, Nigeria in 2011 to validate coverage calculated from treatment registers. A total of 352 households were surveyed from 24 randomly selected communities. Heads of household were interviewed to identify household-level characteristics and predictors of participation. Individual household members were enumerated and those present at the time of interview were asked to report individual participation in the MDA. Responses were verified against the community-drug distributor registration log. Approximately 60% of the sample reported receiving either tetracycline eye ointment or azithromycin for trachoma control. Administrative data on treatment estimated coverage at 76% for the three LGAs. The discrepancy between the coverage estimate from administrative data (calculated by the program) and the survey data suggest that cluster random surveys of MDA provide a useful monitoring tool to validate administrative data on treatment coverage. These data support modification of distributor training and MDA delivery to increase coverage in subsequent rounds of MDA.

## Introduction

Trachoma, caused by infection with the bacterium *Chlamydia trachomatis*, is the leading cause of infectious blindness worldwide [1], [2]. Over time, repeated infections develop scar tissue on the inside of the eyelid, pulling the eyelashes towards the eye where they abrade the cornea, resulting in corneal opacity, low vision and blindness. Where trachoma is a public health problem, the World Health Organization (WHO) recommends the implementation of the full SAFE Strategy (Surgery, Antibiotic Therapy, Facial Cleanliness and Environmental Change). The “A” arm of the SAFE strategy calls for antibiotic treatment with a target of administering antibiotics to at least 80% of the population, using oral azithromycin or topical (ophthalmic) tetracycline ointment which treats individual cases and reduces the reservoir of ocular chlamydia in the community. If the prevalence of clinical trachoma (grade trachomatous inflammation, follicular, known as “TF”) exceeds 10% among children one to nine years of age, mass distribution of antibiotics is warranted at the district level (defined as an administrative unit of approximately 150,000–250,000 persons). Once initiated, district-wide antibiotic distribution is implemented annually until the program reduces the prevalence of clinical signs of trachoma among children to below 10% [3].

Prevalence surveys conducted in Nigeria suggest that trachoma is of public health importance in parts of northern and central Nigeria [4]–[7]. Seven of the 30 LGAs surveyed had a prevalence of TF among children ages one to nine years of age greater than 10%, qualifying for district-wide mass drug administration (MDA) of antibiotics for trachoma control in the context of the full SAFE strategy. In June 2010, Nigeria received its first donation of azithromycin from Pfizer, Inc. via the International Trachoma Initiative for the implementation of district-wide MDA in these seven LGAs. The MDA treatment protocol for trachoma is described elsewhere [8].

The Plateau and Nasarawa state ministries of health implemented the first MDA for trachoma control in the seven eligible LGAs from October to November 2010. It was delivered through the existing community-directed treatment network established in the 1990s to implement MDA for onchocerciasis, and more recently lymphatic filariasis, and schistosomiasis [9]. Under this delivery model, two to four volunteer community drug distributors (CDDs) are selected from the community by local leadership to implement MDA activities. Smaller communities are often served by a CDD from a neighboring village. All communities in the seven trachoma-endemic LGAs were eligible to participate in the MDA and the entire LGA population was targeted for treatment. CDDs participate in annual two-day trainings on the drug distribution protocol to prepare them to register community members, manage drug supply and administer treatment. Prior to MDA, CDDs are responsible for mobilizing community participation via health education sessions. Over the course of a two-week MDA, CDDs administer drugs at central sites in their community or conduct treatments at the household level. CDDs are not compensated directly by the program, but may receive other opportunities to earn *per diem* because of their experience or receive cash or gift-in-kind contribution from their community. Each individual who resides in the community is named in the CDD register. When the community member receives treatment during MDA, his/her participation is recorded by the CDD. Upon completion of the MDA, the number of people treated by each CDD is reported to the LGA and state health services. The administrative coverage of MDA is calculated by aggregating the treatment reports from all CDDs at the LGA-level and dividing by the total population of the LGA as estimated by the MDA registered population.

In other trachoma control programs, management of the drug is the responsibility of the health personnel and stocks of azithromycin are carefully monitored within the health system and not handed over to volunteers or left in the villages overnight. Therefore, to validate coverage estimates calculated from CDD reports, we conducted a survey to estimate the prevalence of participation in trachoma MDA. The primary objective of the study was to estimate the proportion of individuals who participated in the MDA. Secondary outcomes included the proportion of trachoma-related health education among heads of household and the proportion of participation verified by the CDD register.

## Methods

**Figure 1:**
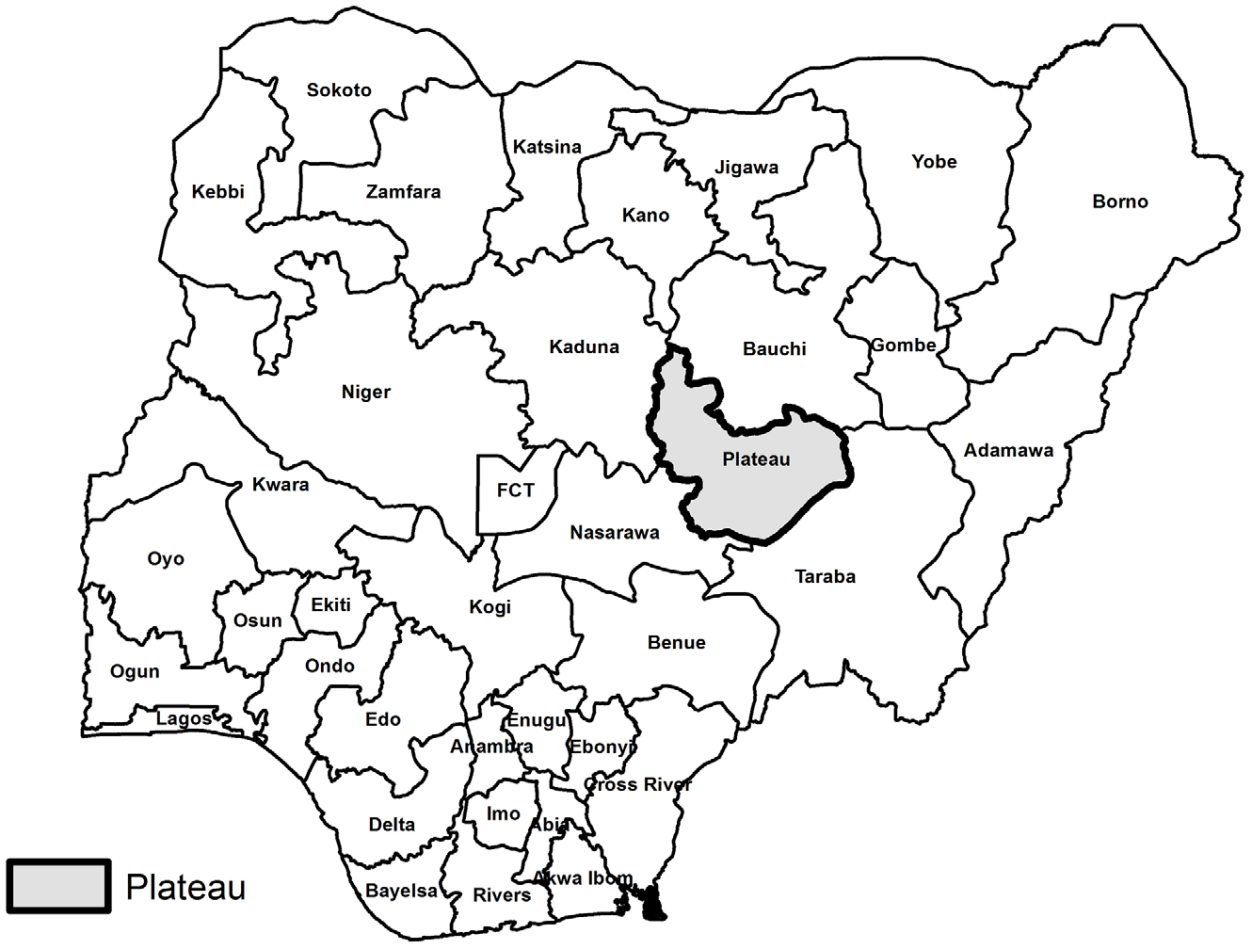
Location of Plateau State, Nigeria.

**Figure 2:**
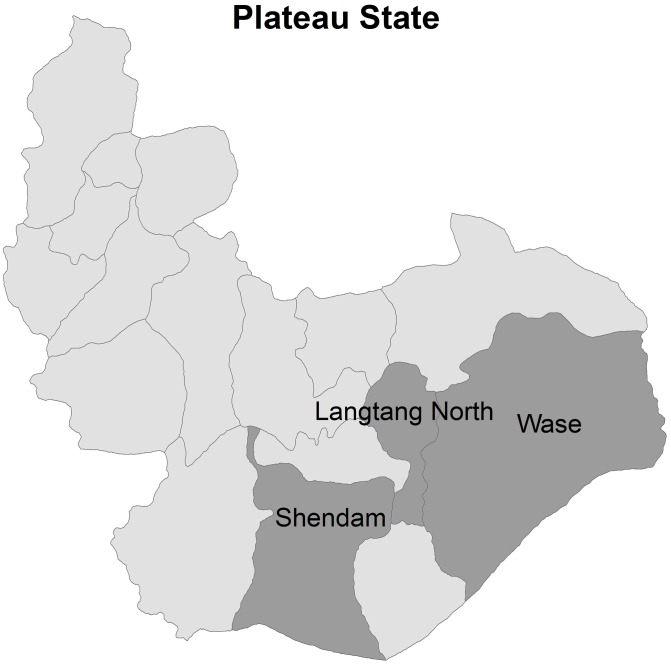
Map of Local Government Areas (LGAs) surveyed.

In January 2011, a two-stage cluster random survey was conducted in three LGAs in Plateau State: Shendam, Langtang North and Wase, as shown in Figure 1 and Figure 2. These LGAs were chosen in order to integrate the survey with ongoing monitoring activities. Since the implementation of MDA for trachoma in the three LGAs began at the same time in October 2010 and employed the same protocol, all three LGAs were surveyed as one domain to generate an overall prevalence estimate of participation in the MDA.

### Sample size

In order to detect a prevalence of participation in MDA of at least 50% with 80% power, and an alpha level of 0.05, including a design effect of 4.0 [5] and a 20% non-response rate, a sample size of 1,843 persons was required. Assuming an average household size of five persons and 24 clusters, 15 households per cluster were planned to be surveyed.

### Study design

This survey was implemented using a two-stage cluster random survey design. Clusters were defined using the census administration unit known as an Enumeration Area (EA), for which borders are well-delineated and populations are approximately the same size, eliminating the need for probability proportional to size sample selection. In the first stage, the total 2,838 mutually exclusive EAs in the three LGAs were listed in order of geographic proximity and 24 clusters were selected systematically by calculating a sampling interval (n), randomly selecting the first EA from the first *n* EAs and the 23 subsequent *n* ^th^ EAs. In the second stage, sampling of households was performed using the map and segment method to ensure equal, non-zero probability of selection within each cluster [10]. Using cluster maps, the cluster was then divided into segments of five households each, and three segments were randomly selected. All households in the selected segments were eligible to participate in the survey. A household was defined as a group of people who ate from the same cooking pot. If two or more households were enumerated in the same dwelling, the individual heads of households were interviewed separately. The survey team returned to vacant households at least twice on the day of interview. Heads of households who were out, made themselves repeatedly absent, or declined to participate were not replaced.

### Data collection methods

In each household, informed consent was obtained from the head of household (or available household member aged 15 years and older) before the interviews began. A standard pre-coded questionnaire was administered to each head of household to collect basic household demographic data and knowledge of trachoma disease, the SAFE strategy and the household-level interaction with the CDD during MDA. The survey teams also reported the physical presence of a household pit latrine and whether there was evidence of use (feces in the pit) as a proxy indicator of the household's participation in hygiene and sanitation programs. After the head of household interview was completed, the members of the entire household (including those absent) were enumerated on a census form. Each member was asked to report their age, type of antibiotic taken (if any), and reason for not participating, if applicable. Children ages 6–15 years of age were asked if they had ever received multiple drug treatment for onchocerciasis, lymphatic filariasis, schistosomiasis, or soil-transmitted helminthes after being shown an example of the pills administered for each disease. In the event that a household member was absent, the head of household responded as a proxy.

Survey participants were shown examples of azithromycin bottles and tablets and empty pediatric oral suspension bottles to enhance recall. Once all household interviews in the cluster were completed, the survey team reviewed the CDD register to verify the responses of the participants. For example, if a participant said that s/he did not receive treatment but the CDD logbook indicated that s/he had, the response would be recorded as “not verified”. If the participant was not listed in the register then the response was coded as “not recorded”.

Data collection team members were recruited from Plateau State and participated in training on the study protocol, household selection, and the administration of the questionnaire. The questionnaire was translated into Hausa and back-translated into English to verify the accuracy of the translation. Before the fieldwork began, the questionnaire was pilot-tested among households in a village not selected for the study.

### Statistical analysis

Survey data were double-entered and validated in Microsoft Access 2003. The analysis was conducted using SAS version 9.2 (The SAS Institute Cary, NC). The primary outcome of the study was the proportion of total respondents who were present during the survey that reported having taken antibiotics during the MDA. The household-level questionnaire was analyzed using the SURVEYFREQ procedure to account for the correlation among the data due to the study design and to allow weighted analysis to adjust for differences in probability of household selection between EAs. All frequencies presented in this paper are weighted unless otherwise specified. Individual level responses from the census form were also analyzed using cluster-level weights. The proportion of individuals who reported participation was calculated using only the individuals present at the time of interview to reduce the introduction of systematic error due to recall and response bias on behalf of other household members. A kappa coefficient was calculated using the proportion of agreement between household responses and the CDD register.

In order to determine if there was an association between age and gender among individual responses and the proportion of participation in the MDA, linear regression using a generalized estimating equation (GEE) with a binomial distribution and identity link was performed to calculate the prevalence difference of participation among individuals whose head of household reported prior knowledge of trachoma MDA, knowledge of the purpose of trachoma MDA and treatment received at home. A sensitivity analysis was performed to compare the probabilities derived from a logistic regression model and the linear regression to check the model fit. Confounding by head of household age, gender, and head of household advance knowledge of the MDA was assessed. The prevalence difference of participation was also calculated for the subset of the households where the head of household reported having received antibiotics for trachoma from a CDD.

Administrative treatment data and population estimates from the MDA were provided by the Plateau State Trachoma Control Program in order to compare the administrative coverage against the results of the survey. Administrative coverage was calculated as the number of people treated with MDA divided by the total population of the LGA.

### Ethical considerations

This study was reviewed and approved by the Emory University IRB under protocol 00009342. The Plateau State Ministry of Health approved the survey as an ongoing program monitoring activity. Upon arrival in the community, standardized consent statements were read in Hausa or the local language to the village head to request permission to enter the community. Verbal informed consent was obtained from the head of household prior to conducting interviews in the household. Each adult household member selected for participation was read a standardized consent form and verbal consent obtained prior to administering the survey, Verbal informed consent was obtained from parents or guardians of children under the age of 18 and verbal assent was obtained from children ages 6 and above prior to conducting interviews. Oral informed consent/assent was approved by the Emory IRB due to low literacy rates in the population and because no samples or specimens were taken during the survey, causing no additional risk to the participants than those experienced in daily life. Oral consent/assent was documented on individual survey forms prior to the commencement of data collection. No incentives were offered or provided to any participant.”

## Results

### Survey responses

The survey team visited 24 EAs, where a total of 392 households were eligible for interview. Verbal informed consent was obtained from 352 heads of households, for a head of household-level response rate of 90%. Figure 2 illustrates the composition of the sample. The weighted proportion of household-level respondents who were male was 61.4% (95% CI: 52.5–70.3%) and the mean age of the head of household respondent was 41.7 years (SD: 1.1). The mean household size was 5.9 persons (SD: 0.2). A pit latrine was observed in 30.4% of households (95% CI: 17.8–42.9%).

The results of household-level responses on participation in trachoma MDA are summarized in Table 1. Knowledge of trachoma was assessed by asking the head of household to name the sequelae of the disease if he/she claimed to know of trachoma. Among those who said they knew of trachoma, 94.1% (95% CI: 91.1–97.1%) demonstrated knowledge of the disease by their survey response and 87.9% (95% CI: 82.2–93.5%) demonstrated knowledge of some aspect of the SAFE strategy at the time of the survey.

**Table 1:** Head of Household Responses (n = 352 unless specified).

Indicator	N	%*	Lower CI^1^	Upper CI
*Trachoma*				
Received Antibiotics from CDD				
Yes	255	69.0	56.3	81.6
No	94	31.0	18.4	43.7
Missing data	3			
Location where drugs administered by CDD (n = 255)				
At a household	238	95.9	88.9	100.0
Outside the household	15	4.1	0.0	11.1
Missing data	2			
Had advance information about MDA				
Yes	254	75.9	65.1	86.6
No	80	24.1	13.4	34.9
Missing data	18			
Source of information about MDA (n = 254)				
CDD	202	76.0	64.4	87.6
Other sources, not CDD	47	24.0	12.4	25.6
Missing data	5			
Aware antibiotics target trachoma				
Yes	192	56.9	43.1	70.8
No	139	43.1	29.2	56.9
Missing data	21			
HoH knowledge of trachoma				
Yes	270	94.1	91.1	97.1
No	19	5.9	2.9	8.9
Missing data	63			
HoH knowledge of SAFE strategy				
Yes	250	87.9	82.2	93.5
No	33	12.1	6.5	17.8
Missing data	69			

* All frequencies are weighted by survey cluster.

1 CI: 95% Confidence Interval.

A total of 2,103 people were enumerated in the household census. Among those enumerated, 1,791 people were present at the time of the survey. The mean proportion of members present for the interview in each household was 89.3% (95% CI: 84.2–94.4%). Among those present, 60.3% reported having received azithromycin or tetracycline eye ointment during MDA (95% CI: 47.9–73.8%). Participation in MDA among children ages 1–9 years (the target age group of the trachoma control program) was 58.8% (95% CI: 42.9–74.6%). Approximately 54.1% of children ages 6–15 years (of a total 492 children) reported ever participating in a prior non-trachoma MDA (95% CI: 35.1–73.1%), with 25.7% (95% CI: 8.3–43.1%) of those respondents able to identify the disease for which they were treated. Table 2 presents the weighted frequencies of participation in the MDA for trachoma control for those present at the interview. The prevalence difference estimates derived from the model are presented in Table 3. Overall, there is a large difference in participation among individuals if the head of household was informed in advance and knew about the purpose of the trachoma MDA.

**Table 2:** Individual Participation in trachoma MDA (N = 1,791).

Indicator	N	%*	95% CI
Gender of respondents			
Male	858	47.7	45.7–49.6
Female	924	52.3	50.4–54.3
Missing data	9		
Age			
<5 years	266	14.6	12.8–16.4
5–9 years	297	15.9	13.2–18.5
10–19 years	421	23.9	22.1–25.8
20–29 years	295	17.1	14.6–19.7
30–39 years	213	11.4	10.1–12.8
40–49 years	135	7.6	6.8–8.4
50–59 years	85	4.8	3.6–5.9
60–69 years	39	2.5	1.7–3.3
70 years and older	40	2.2	1.6–2.8
Participation in trachoma MDA			
No	629	39.6	26.2–53.1
Yes	1072	60.4	46.9–73.8
Missing data	90		
Drug type (n = 1072)			
Azithromycin	997	93.4	87.0–99.9
Tetracycline Eye Ointment	22	1.8	0.6–2.9
Both	53	4.8	0.0–11.1
Verification of treatment among respondents			
Record matched CDD register	658	56.6	31.2–82.1
Record did not match CDD register	24	2.4	0.0–6.5
No record of participant	546	41.0	16.0–66.0
Missing Data	562		
Reason for not participating (n = 629)			
Refused	25	3.5	0.6–6.5
Missed	220	33.7	15.2–52.3
Not distributed	373	62.7	42.8–82.6
Missing data	11		
Ever taken drugs for other MDA^1^ (n = 492)			
No	187	45.9	26.9–64.9
Yes	232	54.1	35.1–73.1
Missing data	73		

* Frequencies are weighted by cluster.

1 Among children 6–15 years of age.

**Table 3:** Prevalence difference estimates.

Effect	Prevalence Difference*	95% CI
Difference in proportion of participants		
Gender of participant (male v female)	0.04	−0.02–0.47
Head of household prior knowledge of MDA	0.22	0.09–0.35
Head of household prior knowledge of MDA (among households reporting MDA from a CDD)	0.03	−0.12–0.18
Head of household reported knowing MDA was for trachoma control	0.36	0.12–0.47

* Calculated with survey weights.

### Verification of CDD registers

**Figure 3:**
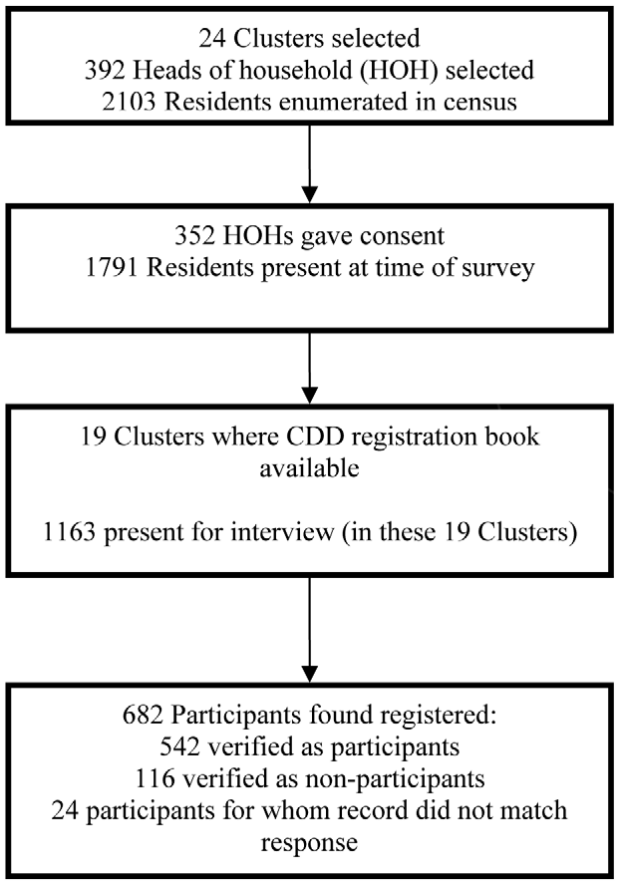
Participation in the survey and verification with CDD registration books.

Comparison of participant responses to the CDD register was only feasible in 19 out of the 24 EAs due to either the absence of the CDD or the register at the time of the survey; all household members for whom verification data were available were included in this sub-analysis. The number of participant records that were verified is described in Figure 3. In the 19 EAs where the CDD register was available, a total of 1,163 people were present for the census. From that sub-group of the study population, a total of 658 participant responses were verified out of 682 responses that were compared against the CDD register: 542 responses were verified as having participated, 116 were verified as having not participated and 24 responses did not match the record in the registration book. Of the responses that could be verified, agreement with the register was 96% (632/658, unweighted), with a kappa coefficient of 0.88 (95% CI: 0.84–0.93). Among the entire study population present at the time of interview, 41.4% (481/1,163, unweighted) were not listed in the CDD register book (where a register book was examined for that cluster during the survey). To estimate the proportion of participation in the MDA among the clusters where the CDD registers were employed, we restricted the analysis to responses that were verified by the CDD register book. In these clusters, 79.9% (weighted for cluster) of the respondents participated in the MDA (95% CI: 76.9–82.9%).

### Administrative data

The Plateau State Trachoma Control Program reported administrative coverage of MDA as 75.8% overall, being: 112,066 persons treated in Langtang North (80% of the 140,643 total population); 185,454 persons treated in Shendam (89% of the 208,017 total population); and 89,166 persons treated in Wase (55% of the 161,714 total population). This compares to 60.3% overall coverage reported by participants randomly selected from all LGAs in the cluster random.

## Discussion

The results of this coverage survey and the administrative data from the MOH show that the population coverage of MDA did not meet the WHO-recommended minimum target of 80% coverage. The overall proportion of the three LGA-level coverage estimates from the MOH administrative data (75.8%) overstates the coverage obtained in these areas compared to the survey point estimate of 60.3%. Participation among children ages 1–9 years (58.8%) was similar to the overall survey estimate. Participation among clusters where treatment was correctly recorded in the registers (79.9%) was similar to the administrative data, suggesting that coverage was greater were the CDD followed MDA protocol. The proportion of children ages 6–15 years who recalled participating in non-trachoma MDA was approximately 54.1%, which is consistent with the coverage estimated for trachoma MDA.

This survey compared CDD registers with participant survey responses as a novel method to verify responses post distribution and quantify recall bias. Although verification of responses was not possible in all clusters, the high concordance of responses (96%) with the treatment registers suggests that participant recall was accurate. Participants were also shown examples of the antibiotic treatments to enhance recall and avoid confusion with non-trachoma MDA treatments (which differ markedly in shape, size and color of tablets and are administered separately). Although agreement between respondents and CDD registers was high, the proportion of responses that were not verified suggests that use of CDD registers could be strengthened through additional CDD training.

Almost a third of head of household respondents reported receiving antibiotics from someone other than a CDD (refer to Table 1), suggesting that the community may not be universally aware of the identity of the CDD or the CDD works with other community members to administer treatment. Since MDA in Nigeria is community-directed, these data support the recommendation that future CDD training activities address the role of community volunteers in pre-MDA mobilization. Reported knowledge of trachoma was high among head of household respondents, yet only 56.9% of respondents knew what azithromycin and tetracycline treated. This suggests that although awareness of the SAFE strategy is prevalent, knowledge of MDA for trachoma should be reinforced by the CDD during mobilization activities. Knowledge of the diseases for which the other MDA drugs are distributed was also comparably low among children ages 6–15 years, further reinforcing the need for additional health education either before or during MDA for all control programs in Plateau state.

Although these findings are sufficient to generate recommendations for future implementation of MDA, the results of the survey should be interpreted in light of the following limitations. The three LGAs were sampled as one evaluation unit, which does not estimate population coverage for the individual-level LGAs. As illustrated by the administrative data, there are likely to be minor variations in the coverage estimates and household-level characteristics at the LGA-level. We calculated frequencies without correcting for household-level correlation; however, a sensitivity analysis comparing the results from PROC SURVEYFREQ and a GEE controlling for household showed that the results from these approaches were similar. The administrative unit for the clusters, the census EA, did not always match exactly the catchment areas for individual CDDs, which may have introduced bias in terms of verification of responses with CDD registers. Although the head of household-level response rate was almost 90%, missing data from absent individuals reduces the sample size and precision in our estimates. Notably, the missing CDD registers limit the generalizability of the kappa statistic.

The implementation of a cluster random survey to estimate the population coverage of MDA enables trachoma control programs to identify opportunities to improve the implementation of MDA and community participation. Without periodic coverage surveys, programs would be reliant on administrative coverage estimates to monitor treatment performance. The discrepancy between reported administrative coverage and the survey results in this evaluation suggest that reliance on administrative data alone is not sufficient for monitoring of MDA performance. In other settings, cluster random surveys to estimate the population coverage of mass distribution interventions have shown that administrative records are not always reliable [11]–[13]. The low coverage achieved by this MDA may be attributed to the use of village lists for planning and implementation of the program from other disease control programs, whereas in clusters where the CDDs followed program monitoring guidelines using treatment registers, the survey results are more consistent with administrative data. To improve the performance of future antibiotic distributions, these data suggest that there is a need to review community-level population estimates to ensure that coverage calculated from CDD registers is accurate. Furthermore, if the program population data underestimate the true population, administrative coverage data will be inflated, and the supply of future antibiotics may not be sufficient to treat the entire population.

Administrative data is often biased due to a reliance on population estimates for the denominator, which can be incorrect between census years. Administrative data is also vulnerable to bias from lost forms or inaccurate records. Conducting a cross-sectional survey to measure the prevalence of participation in MDA provides an opportunity for trachoma control programs to validate the administrative reports and measure covariates to identify factors associated with increased participation. Accurate population coverage estimates are essential to measuring the overall effectiveness of the SAFE strategy on reducing trachoma as part of routine program evaluations. These data demonstrate that household surveys, when compared with administrative coverage data, are a useful tool to monitor MDA performance.
